# Death of Dr. James Ives

**Published:** 1879-07

**Authors:** 


We learn with sorrow of the death of Dr. JAMES IVES, of Strykersville, Wyoming county, N. Y. Dr. IVES was an experienced and capable physician of the old stamp, was in high estimation by his professional associates and by the community in which he had lived so long and labored so faithfully. In his later years he had relinquished much of his business which in former years had been extensive and very laborious. His associate physicians will feel his death a great loss in their consultations, while his patrons and friends will long remember his earnest, faithful ministrations in times of trial and affliction.

